# Prediction of Severity of Drug-Drug Interactions Caused by Enzyme Inhibition and Activation

**DOI:** 10.3390/molecules24213955

**Published:** 2019-10-31

**Authors:** Alexander Dmitriev, Dmitry Filimonov, Alexey Lagunin, Dmitry Karasev, Pavel Pogodin, Anastasiya Rudik, Vladimir Poroikov

**Affiliations:** 1 Institute of Biomedical Chemistry, 10 Building 8, Pogodinskaya Street, 119121 Moscow, Russia; dmitry.filimonov@ibmc.msk.ru (D.F.); alexey.lagunin@ibmc.msk.ru (A.L.); w.dmitrykarasev@gmail.com (D.K.); pogodinpv@gmail.com (P.P.); rudik_anastassia@mail.ru (A.R.); vladimir.poroikov@ibmc.msk.ru (V.P.); 2 Department of Bioinformatics, Pirogov Russian National Research Medical University, 1 Ostrovityanova Str., 117997 Moscow, Russia

**Keywords:** drug interactions, DDIs, adverse drug reaction, ADR

## Abstract

Drug-drug interactions (DDIs) severity assessment is a crucial problem because polypharmacy is increasingly common in modern medical practice. Many DDIs are caused by alterations of the plasma concentrations of one drug due to another drug inhibiting and/or inducing the metabolism or transporter-mediated disposition of the victim drug. Accurate assessment of clinically relevant DDIs for novel drug candidates represents one of the significant tasks of contemporary drug research and development and is important for practicing physicians. This work is a development of our previous investigations and aimed to create a model for the severity of DDIs prediction. PASS program and PoSMNA descriptors were implemented for prediction of all five classes of DDIs severity according to OpeRational ClassificAtion (ORCA) system: contraindicated (class 1), provisionally contraindicated (class 2), conditional (class 3), minimal risk (class 4), no interaction (class 5). Prediction can be carried out both for known drugs and for new, not yet synthesized substances using only their structural formulas. Created model provides an assessment of DDIs severity by prediction of different ORCA classes from the first most dangerous class to the fifth class when DDIs do not take place in the human organism. The average accuracy of DDIs class prediction is about 0.75.

## 1. Introduction

Many drug-drug interactions (DDIs) are caused by alterations of the plasma concentrations of one drug (victim or object drug) due to another drug (precipitant drug). Precipitant drug inhibits and/or inducts (activates) the metabolism or transporter-mediated disposition of the victim drug. Some DDIs can cause more than tenfold increase or decrease in a victim drug exposure, with life-threatening consequences. Thus, predictions and accurate assessment of clinically relevant DDIs for novel drug candidates represent one of the significant tasks of contemporary drug research and development [1,2,3]. According to the requirements of US Food and Drug Administration (FDA) and European Medicines Evaluation Agency (EMEA), it is essential to determine DDIs as early as possible during the drug discovery and development process [1]. Usually, at the final stages of the costly drug discovery cycle, the pharmaceutical companies prioritize the list of potential DDIs, which must be checked. Then investigators select appropriate in vitro assays, then in vivo studies, optimize the design of experimental testing and perform clinical trials. However, such a strategy can be dangerous because the developed new chemical entity (NCE) may finally be unacceptable due to the DDIs found in experiments. DDIs can lead to life-threatening consequences and a recall of newly developed drugs from the market. The examples of such recalled drugs are Mibefradil, Cisapride, Terfenadine, Astemizole [4]. Computational (in silico) methods that predict DDIs can be used to avoid such dire consequences. A comprehensive description of the methods for DDIs prediction is presented in the review [3]. There are computational methods that predict DDIs indirectly, for example, ligand-based or structure-based methods dealing with substrates, inhibitors, and inducers of drug-metabolizing enzymes (DMEs) [5,6,7]. Prediction results of these methods can be used to obtain a conclusion about possible DDIs. Methods for direct estimation of DDIs include literature-based DDIs prediction using medical records [8] and methods based on structure resemblance, and functional similarities [9,10,11]. However, the results of such DDIs prediction are typically provided without the assessment of importance and severity of DDIs manifestation. Very often, the results of prediction obtained by these methods are given in databases that contain a conglomeration of information about predicted DDIs between drugs without assessment of the severity of possible DDIs manifestation [12,13].

We have earlier created PASS (Prediction of Activity Spectra for Substances) [14] and GUSAR (General Unrestricted Structure-Activity Relationships) software that use structural formulas of compounds and special mathematical algorithms to predict various biological and pharmaceutical properties of compounds. With the use of PASS and GUSAR, a wide diversity of computational experiments concerning drug metabolism and DMEs were carried out [15], and a dozen of freely available web services were created [16]. We developed a model based on the GUSAR program for the prediction of DDIs directly using combined descriptors for a pair of substances involved in DDIs [17]. This prediction was performed without assessment of DDIs severity. In our recent work, we created a new method for prediction of DDIs that are associated with the risk to health, based on the PASS algorithm [18]. For that purpose, we used novel Pairs of Substances Multilevel Neighbourhoods of Atoms (PoSMNA) descriptors, which take into account structures of two potentially interacting molecules. The model was created using the training set with drug pairs with known DDIs classes assigned according to OpeRational ClassificAtion (ORCA) system. The created model allows predicting not only the fact of DDIs but also the severity of DDIs manifestation for three out of five ORCA DDIs most dangerous classes. The developed method was used for Abiraterone, Erythromycin and cytochrome P450 3A4 interaction prediction [19]. A special web resource for DDIs prediction (http://way2drug.com/ddi/) was created based on the developed SAR (Structure-Activity Relationships) models.

The current study is dedicated to creating SAR model that will be able to predict not only three but all five ORCA DDIs severity classes: Contraindicated (Class 1), provisionally contraindicated (Class 2), conditional (Class 3), minimal risk (class 4), no interaction (Class 5). The model is based on the same approach as in our previous research [18]; however, it contains the new training set data on five ORCA classes, as opposed to three ORCA classes used in the previous research.

## 2. Results

The accuracy of biological activity prediction in the standard PASS version is estimated by the leave-one-out cross-validation (LOO CV) procedure with a calculation of Invariant Accuracy of Prediction (IAP) parameter. To estimate the accuracy of the created computational model for ORCA DDIs classes prediction, the LOO CV procedure was performed with a modified calculation algorithm. Experimental DDIs classes for a particular pair of compounds are compared with the predicted classes after excluding from the training set, not only this particular pair of compounds. All pairs containing any of the compounds of the estimated pair (see Table 1, “DDIs IAP” column) were excluded. It is called “compound out” strategy of validation [18]. Additionally, 20-fold CV procedure using IAP criterion was performed (see Table 1, “DDIs IAP 20-Fold” column).

DDIs IAP estimation of DDIs prediction accuracy was obtained under conditions when both structures from the estimated DDIs pair are completely excluded from the data set during the training. Thus, such estimation can be called ‘true predictivity’. With this in mind, the obtained accuracy (average DDIs IAP is 0.754, and DDIs IAP 20-Fold is 0.751) appropriates for the application of the model in the assessment of DDIs in practice. A small difference between DDIs IAP and DDIs IAP 20-Fold values displays the robustness of the models. It is important to stress that prediction of the most dangerous class 1 (the contraindicated combination of compounds) and class 2 (provisionally contraindicated) ORCA DDIs classes can be performed with the best IAP 0.876 and 0.788 respectively. Accuracy of class 5 (when no interaction between compounds occurred) prediction is 0.752 that is high enough, which is important too, because the model can distinguish the really occurred DDIs from opposite cases. Accuracy of prediction of boundary classes 3 (conditional) and 4 (minimal risk of combination) between the most dangerous classes 1 and 2 and inactive class 5 is not high enough (0.683 and 0.671 respectively). This low accuracy may be explained by unclear separation in training set of the DDIs of these classes among themselves and with the cases of DDIs in neighboring classes.

## 3. Discussion

The DDIs is a crucial problem because polypharmacy is increasingly common in modern medical practice [20] and the risk of adverse drug reaction (ADRs) caused by DDIs is about 13% for simultaneously used drugs pair, about 58%—for five drugs, and 82%—for seven or more drugs [21]. Assessment of DDIs severity is very important—based on this information researchers in the pharmaceutical chemistry field make conclusions about NCEs perspectives and practical physicians rely on assessment of DDIs severity as it pertains to the strategy and consequences of polypharmacy. Critical problem of such computer models is DDIs overprediction, which leads to false-positive results, because in many cases these computational models do not contain negative examples of DDIs, viz. the pairs of the molecules that do not interact when co-administered in the human organism. To some extent it occurs due to the use by researchers of the freeware DrugBank (https://www.drugbank.ca/) as a source of information for training sets for creation of computer models for DDIs prediction [3], whereas DrugBank does not contain a structured description of DDIs severity. In vivo and in vitro experiments can provide information about negative examples, but the results of such investigation are incredibly scarce. Thus, computational prediction of drugs non-interaction, or insignificant and non health-threatening DDIs, is necessary but has not been realized yet.

The proposed approach for the prediction of ORCA DDIs classes is advanced in comparison with the methods proposed earlier. It is essential that the prediction can be carried out for new (virtual and not yet synthesized) molecules using only their structural formulas. Such assessment is important for NCE development. Another unique feature of the created computational model is that prediction provides an assessment of DDIs severity by estimation of different ORCA classes from the first most dangerous class to the fifth class when DDIs do not take place in the human organism.

The prediction that substrates will not interact, is another very important distinctive feature of the presented method. When creating negative examples for computational models, one must proceed from the results of studies indicating that the substances do not interact (such experimental information is extremely scarce), or from the assumption that pairs of substances, for which DDIs are not described, will not exhibit DDIs (such approach is controversial in case of DDIs). Information source for training set creation that was used in this investigation already contains those negative examples described as the drug pairs that do not interact (class 5). Application of this information in the model for prediction of fifth DDIs ORCA class overcame the problem of negative examples’ deficiency, which is very often observed in QSAR modeling.

Comprehensive “ideal” training set for DDIs prediction for the pair of compounds should contain a description of all possible pairwise combinations of drugs. For example, for 767 drugs, “ideal” training set theoretically should contain information about severity classes for 293,761 pairwise DDIs. However, at present, it is physically impossible to reach this level of data completeness due to the DDIs assessment being the result of human in vivo clinical trials and observations for this great amount of pairs.

The average accuracy of prediction (DDIs IAP = 0.754) is acceptable but not high enough. It can be explained by incompleteness of the training set (see Section 4.2) because only small fraction of all possible pair combinations for 767 drugs is presented in the training set. The training set includes 2090 pairs of drugs. It is less than one percent (0.71%) of all 293,761 possible pair combinations of 767 drugs. The rest 291,671 pairs are not described in the book due to the lack of any such information available in publications for the reason that these combinations have not been experimentally evaluated yet, or the negative results of experiments (lack of DDIs for such combinations) have not been published. Another possible problem with the data set is the absence of clear boundaries between the classes, especially between the third and fourth DDIs classes, because it is clear that the difference between "conditional risk" (Class 3) and "minimal risk" (Class 4) is very fuzzy. This blurring between the classes affected the model that we can see for the third (IAP = 0.68) and the fourth (IAP = 0.67) classes. However, the accuracy obtained for marginal cases of DDIs Class 1 (IAP = 0.876) and Class 5 (IAP = 0.752) is higher. The prediction that the substances definitely should not be taken together (Class 1) or the prediction that the substances are safe when taken together (Class 5) is more important than the assumption about minimal (Class 4) or conditional (Class 3) risk. It should be noted that the prediction of the second class of DDIs (provisionally contraindicated) may be performed with a good accuracy (IAP = 0.79). Thus, the model, even with such an imperfect training set, is useful. Further progress in increasing the accuracy of the assessment will be achieved by improving, refining, and expanding of the training set.

## 4. Materials and Methods

### 4.1. OpeRational ClassificAtion (ORCA) System

The number of potential DDIs that can be predicted by in silico methods, is large and could contain a lot of false-positive results. Many of these predicted DDIs can also be detected in the human organism but are not clinically relevant; therefore, it is essential to assess the severity of predicted DDIs. The DDIs severity can be defined by applying different classification systems based, for example, on the "clinical significance" of levels of documentation. In our previous [18] and present investigations, we applied the OpeRational ClassificAtion (ORCA) system developed and provided by The Drug Interaction Foundation for the classification of DDIs [22]. ORCA system was created for clinicians to assess the potential risk of drug pairs co-administration. ORCA assigns the pairs of drugs into five categories based on the DDIs management: contraindicated (Class 1), provisionally contraindicated (Class 2), conditional (Class 3), minimal risk (Class 4), no interaction (Class 5). Belonging to one of the classes means explicit recommendations for clinicians. Belonging of the pair of compounds to the first ORCA class means that "no situations have been identified where the benefit of the combination outweighs the risk." Belonging to the second ORCA class means that "the combination increases the risk of adverse effects; avoid concurrent use unless interaction is desired or no alternative is available; if the combination is used, increased monitoring may be necessary." In the case of the third class, it is noted that "risk may be increased, depending on the clinical situation; assess risk and take action as needed." The fourth class means that "the risk of adverse outcome appears small, and no special precautions appear necessary." Finally, the fifth ORCA class for the pair of substances means that" evidence suggests that drugs do not interact" [22].

### 4.2. Training Set Creation

Hansten and Horn’s Drug Interaction and Management 2013, published by Philip D. Hansten and John R. Horn [23] was the source of information for the creation of a training set for in silico prediction of the severity of DDIs. The information contained in this book is derived from the analysis of literature after it was reviewed and approved by the authors. Interacting drugs are unambiguously assigned to one of five ORCA classes. Management options are given for each described in book interacted pair to offer the clinicians the actions that may be taken to reduce the risk of adverse outcomes. All the cases of DDIs specified in the book belong to the first, second, third, fourth, and fifth ORCA classes. For the training set creation, we collected data for 767 drugs’ structural formulas of 2090 pairs described in this book. These pairs belong to class 1 (59), class 2 (236), class 3 (1139), class 4 (523), and class 5 (133) of DDIs. Structural formulas of 767 molecules of drugs were collected and represented in MOL format. The table of pair DDIs for these 767 drugs contains an indication of DDIs severity classes for all 2090 pairs, but it is clear that this amount of known DDIs is only a small part of all 293,761 possible pair combinations for 767 molecules.

### 4.3. PASS

The PASS software [14] predicts the profile of biological activity based on advanced naïve Bayes classifier. Input data for the algorithm are the structural formulas of drug-like organic compounds in MOL format. PASS output is a ranked list of activities with probabilities ”to be active“ Pa and ”to be inactive“ Pi. PASS prediction algorithm is based on the analysis of structure-activity relationships (SAR) for the training set of compounds with known biological activities. Currently, PASS predicts over 5000 kinds of biological activity, including pharmacological effects, mechanisms of action, toxic and adverse effects, interaction with metabolic enzymes and transporters, influence on gene expression, etc. Biological activities are represented qualitatively as “active” or “inactive” in the PASS program. The molecular structures of drug-like organic compounds are described by Multilevel Neighbourhoods of Atoms (MNA) descriptors. Accuracy of prediction is estimated in PASS by LOO CV and 20-fold cross-validation procedures using IAP criterion. IAP is a sample estimate of the probability that randomly selected from an independent test set positive and negative examples will be correctly classified; IAP coincides with AUC value [14]. 

DDIs estimation by PASS is similar to the biological activity prediction procedure, but for DDIs prediction, the input data represented not by single molecules but by the pairs of structural formulas of compounds. The results of prediction for each pair of compounds are represented by the lists of five DDIs ORCA classes. For each ORCA class in the list two probabilities Pa (probability of belonging to a particular ORCA class) and Pi (in the opposite case) are calculated.

### 4.4. Pairs of Substances Multilevel Neighbourhoods of Atoms Descriptors

**Figure 1:**
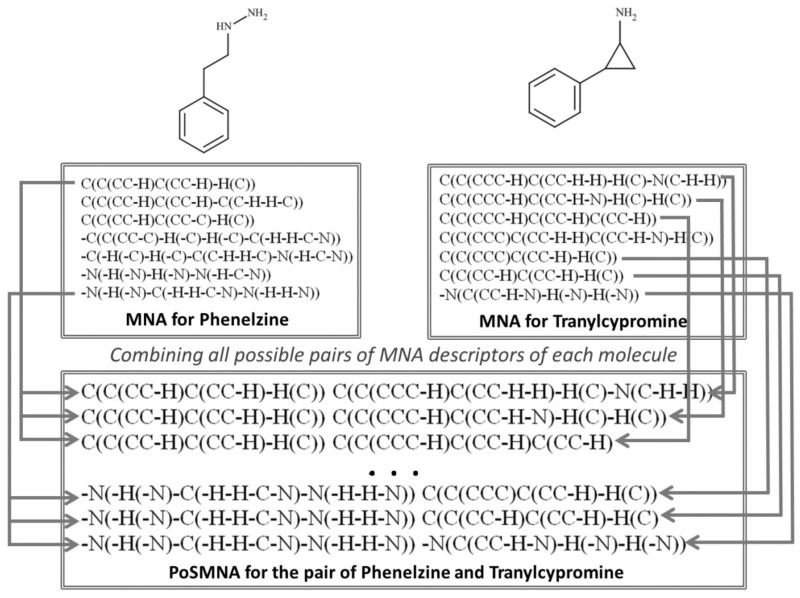
Representation of the Phenelzine and Tranylcypromine molecules by MNA and PoSMNA descriptors.

In our previous work we developed PoSMNA descriptors [18]. PoSMNA can be used for the prediction of various phenomena where pairs of molecules act together, e.g., for prediction of DDIs or prediction of synergetic effects of two drugs. In this investigation, PoSMNA descriptors were used in PASS to describe the structures of drug pairs instead of Multilevel Neighborhoods of Atom (MNA) descriptors used for single molecules in the standard version of PASS program [14]. The pairs of the substances are represented by the PoSMNA descriptors. PoSMNA is a set of all possible pairs of MNA descriptors of each of the molecular pair as strings of symbols ”MNA descriptor of the first molecule“, ”space“, ”MNA descriptor of the second molecule“. Second level MNA descriptors (MNA/2) for heavy (not hydrogen) atoms were used for PoSMNA assembling. In each pair the MNA descriptors are ordered lexicographically, for example, ”C(C(CCC-H)C(CC-H-H)-H(C)-N(C-H-H)) C(C(CC-H)C(CC-H)-H(C))”, ”C(C(CCC-H)C(CCH-H)-H(C)-N(C-H-H)) C(C(CC-H)C(CC-H) C(C-H-H-C))”, etc. (see PoSMNA descriptors examples for Phenelzine and Tranylcypromine in Figure 1). To create the models for DDIs prediction, PoSMNA descriptors were generated for all pairs of compounds with known classes of DDIs severity (from class 1 to class 5) in the training set.

## Figures and Tables

**Table 1:** Accuracy of the ORCA DDIs classes prediction estimated by the LOO CV and 20-fold CV procedures.

DDIs Class	N	DDIs IAP	DDIs IAP 20-Fold
Class 1	59	0.876	0.876
Class 2	236	0.788	0.777
Class 3	1139	0.683	0.679
Class 4	523	0.671	0.668
Class 5	133	0.752	0.754
**Average**		**0.754**	**0.751**

**N** is the number of drug pairs in the training set belonging to the appropriate ORCA classes; **DDIs IAP** is the invariant accuracy of prediction, obtained by the LOO CV procedure with exclusion from the training set of all the pairs containing one of the compounds of the pair under estimation; **DDIs IAP 20-Fold** is the invariant accuracy of prediction, obtained by 20-fold cross-validation procedures.
